# Dental Erosion and Its Relation to Potential Influencing Factors among 12-year-old Hungarian Schoolchildren

**DOI:** 10.3290/j.ohpd.b2805391

**Published:** 2022-03-14

**Authors:** Máté Jász, Judit Szőke

**Affiliations:** a Assistant Lecturer, Department of Prosthodontics, Faculty of Dentistry Semmelweis University, Budapest, Hungary. Performed the experiment in partial fulfillment of requirements for a doctoral degree, wrote the manuscript.; b Assistant Professor, Faculty of Dentistry, Semmelweis University of Medicine, Budapest, Hungary. Idea, study design, wrote and proofread the manuscript.

**Keywords:** dental erosion, national survey, prevalence, risk factors, 12-year-old-children

## Abstract

**Purpose:**

This cross-sectional observational study evaluated the frequency of dental erosion in 12-year-old schoolchildren in Hungary and its connection to gender, geographical region, eating/drinking habits, and to socioeconomic factors, such as the educational level of their mothers.

**Materials and Methods:**

579 randomly selected children aged 12 (287 boys and 292 girls) were examined in our cross-sectional study from 14 different regions in Hungary. Clinical examinations were carried out by the same examiner, using the ‘Basic Erosive Wear Examination’ (BEWE) index. A self-administered questionnaire was filled in by each child, surveying their oral hygiene, nutritional habits and socioeconomic status.

**Results:**

21.2% of the children showed dentitions with signs of erosion. We found statistically significantly higher BEWE scores in urban than in rural areas (p = 0.0058). There was no difference between genders. Among children drinking carbonated soft drinks once or more daily, the prevalence of BEWE score < 3 was statistically significantly lower than among those who consumed these kinds of beverages less frequently (83.6% vs 90%, respectively, p = 0.034). Children of mothers with a highschool diploma had a BEWE score ≥ 3 statistically significantly less frequently than those whose mothers had not graduated from highschool (8.4% vs 22.5%, respectively, p = 0.000).

**Conclusions:**

The prevalence of dental erosion among 12-year-old children in Hungary is not as high as reported previously in Western European countries. A positive correlation was observed between the consumption of carbonated soft drinks, the educational level of the mothers and the level of erosion. These factors statistically significantly affected the prevalence and severity of erosive dental lesions.

Tooth wear, especially dental erosion, is a growing problem all over the world, especially in industrial countries. This is true for both children and adults. The prevalence of dental erosion, according to surveys conducted in Europe, North America, Africa and Asia, ranges from 6% to 50% in preschool children and 11% to 100% in adolescents (9- to 17-year-olds).^10^ Preschool children show erosion on deciduous teeth, and young schoolchildren already have erosive lesions on permanent teeth. There is a trend towards a more pronounced rate of erosion in younger ages.^1,3,4,5,7,9, 11,12,13,14,17,18,20,21^ Therefore, it is important to detect patients at risk early to initiate adequate preventive measures. The increased prevalence and severity of dental erosion is associated with several factors. The most important of these is the rising trend in the consumption of acidic drinks and foods^5,7,9,10,11,13,17,20,21^ and gastro-oesophageal reflux disease.^16^

Dental erosion is probably a growing problem in Hungary as well. However, until now, no national epidemiological erosion examinations have been carried out in Hungary either in children or in adults. The main objectives of the present study were therefore to investigate the prevalence, severity and patterns of dental erosion among 12-year-old schoolchildren and to compare the Hungarian erosive tooth-wear data to those of other countries. Additionally, we aimed to determine the relationship between erosion and dietary habits as well as socioeconomic factors in Hungary. We hypothesised that the prevalence of erosion is approximately the European average.

## Materials and Methods

This study is based on a cross-sectional collection of epidemiological data, gathered between February 2014 and June 2015.

**Fig 1:**
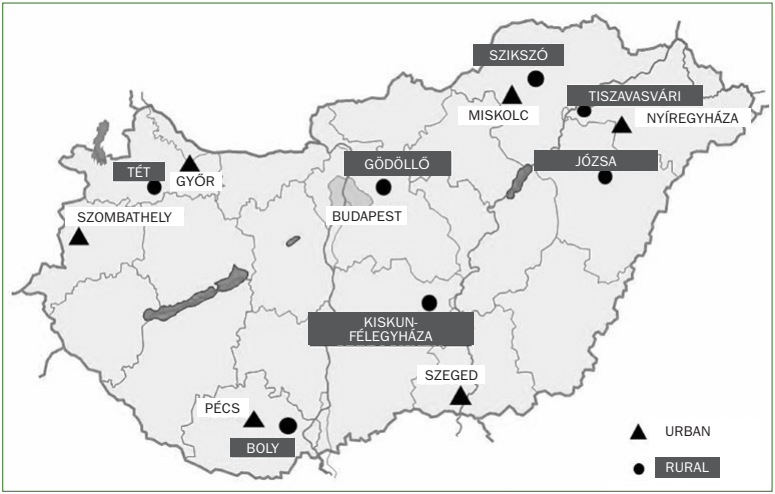
Urban areas: Budapest (capital), Győr, Pécs, Szombathely, Szeged, Miskolc and Nyiregyháza. Rural areas: Gödöllő, Tét, Boly, Szikszó, Józsa, Tiszavasvári and Kiskunfélegyháza.

For such studies, the World Health Organization (WHO) recommends a practical, economic survey-sampling methodology, called the ‘pathfinder’ method.^19^ The WHO suggests data collection from different points in the country’s capital, as well as in at least two large towns and four villages in rural areas as a minimum, but for more reliable final results, more sampling sites are advised. We collected data at 14 sites: seven urban, of which one is the capital, Budapest, and seven rural areas.^18^ The data from three different sampling points in the capital were pooled. The different sites cover different geographical and socioeconomic areas of Hungary. The sampling sites were the same as those identified for previous WHO pathfinder surveys (Fig 1).^15^

At each site, from randomly selected schools, 12-year-old children were randomly selected on the occasion of their obligatory school dentistry check-up. In Hungary, parents have the right to refuse consent to this examination, in which case the children would have been excluded from the study. Exclusion criteria included wearing fixed orthodontic appliances, a large amount of plaque or calculus, dental injury or insufficient cooperation. The present study was performed in accordance with the principles of the Declaration of Helsinki and approved by the Regional Ethics Committee (Egészségügyi Tudományos Tanács Tudományos és Kutatásetikai Bizottság ETT TUKEB 4407-0/2011-EKU), Budapest, Hungary.

Following the ‘WHO Oral Health Surveys: Basic Methods’,^19^ we examined at least 25 randomly selected participants at each site, except one: Szombathely.^19^ A total of 609 children were examined, with nearly equal distribution of boys (304) and girls (305). At the end of the study, 30 children were excluded from the final evaluation as per the exclusion criteria mentioned above. The final study population consisted of 579 children (287 boys and 292 girls), 289 of them from urban and 290 from rural areas. The number of children participating in the questionnaire study varied according to the valid number of answers (405 or 556) to each question. Not all of the questions were answered by all of the participants.

### Dental Examinations

Dental examinations were performed in association with the obligatory yearly school dental check-up in the respective school dental office. All participants were brought to the examination site by teachers or study staff. Dental chair, dental mirror and dental operatory light were used. After air drying the teeth, the procedure consisted of visual-tactile examinations. The dental examination was carried out by the same, well-trained dentist on each site, in each case. We performed duplicate examinations to assess intra-examiner error. Two randomly selected children were re-examined at each sampling site. The examiner did not know that the subject had been examined previously. A local schoolteacher was asked to arrange the re-examinations of the children after 30 to 60 min. The second registration in this series was a duplicate, which was not involved in the final statistical analysis. Weighted Kappa scores ranged from 0.89 to 0.92 for intra-examiner reliability.

The Basic Erosive Wear Examination (BEWE) index was used in the present investigation.^2^ This four-level index scores the appearance and severity of erosion on tooth surfaces, recording the most severely affected surface in each sextant, and the sum of these highest scores of the six sextants is the cumulative BEWE score. This index was first described for adults, but it is also suitable for children. To date, not many studies using BEWE are available in this age group. The first permanent molars and the incisors were the only teeth included in this examination because of the “eruption timetable” of permanent teeth. These teeth would have spent at least two years in the mouth after eruption in this age group. Altogether, we examined twelve teeth (16, 12, 11, 21, 22, 26, 36, 32, 31, 41, 42, 46), three surfaces (buccal, occlusal, lingual/palatal) on the molars, and two (buccal, lingual/palatal) on the incisors. Otherwise, the BEWE scoring instructions were followed.^2^ The BEWE index is not only a diagnostic tool, but also gives suggestions if further treatment is needed. A BEWE score of 0–2 means no risk of erosion, the suggested treatment in this group is ‘routine maintenance and observation, repeated at 3-year intervals’. A BEWE score between 3 and 8 means low risk of erosion, and the suggested treatment is ‘oral hygiene and dietary assessment, and advice, routine maintenance and observation, repeated at 2-year intervals’. For the medium (9–13) and high (> 13) risk patients, there are different therapeutic suggestions.^2^

After the examination, special dietary and oral hygiene instructions were given to the examined children.

### Questionnaire

An anonymous, self-administered standard questionnaire for children, recommended by the WHO,^19^ was used in our study. The questionnaire was designed to evaluate commonly consumed acidic foods and drinks, and the amounts and frequencies of intake, oral hygiene habits, as well as the educational level of the mother. In each case there was the possibility to give the answer ‘I don’t know/I don’t remember’. These answers were excluded from the statistical analysis. In some cases, when the child was uncertain about the answer about her/his mother’s educational level, we tried to help her/him to find the correct one asking about the parent’s occupation. Only 552 questionnaires had valid answers regarding the dietary habits and 405 pertaining to the mothers’ educational level; in the rest of the questionnaires, there was not any unambiguous answer.

Based on the recommendation of BEWE scores, the participants were divided into four groups based on the recommended treatment: 1. cumulative BEWE score 2 or less (no risk of erosion); 2. between 3 and 8 (low risk of erosion); 3. cumulative BEWE score between 9 and 13 (medium risk); 4. cumulative BEWE score above 13 (high risk). The answers of the questionnaire were compared and analysed statistically between these groups.

### Statistical Analysis

Tooth surface loss data were summarised as means, SD and prevalence rates. To reveal potential correlations, 2-sided Fisher’s Exact Test and Pearson’s chi-squared test were used, if evaluating two or more dimensions, respectively. The probability level for significance was set at p < 0.05. All statistical calculations were performed using IBM SPSS Statistics for Windows version 24.0 (IBM; Armonk, NY, USA). Missing or ambiguous answers on the questionnaire were excluded from the statistical analysis.

## Results

### Clinical Results

The overall prevalence of erosion was 21.2%. There was no statistically significant difference between genders: 20.9% of boys and 21.6% of girls had evidence of erosive defects at various clinical levels.

The mean BEWE score collected from 14 sample sites in Hungary was 0.39 ± 0.83 (mean ± SD). No statistically significant differences in the mean values were found between girls and boys (0.36 and 0.42. respectively).

The distribution of BEWE scores by geographic location are depicted in Table 1. There were relatively large differences by location type. The highest mean BEWE score was in Szombathely (1.21 ± 1.31), in the most western part of Hungary. The lowest score was detected in the eastern part of the country, in Tiszavasvári (0.039 ± 0.28). In the urban areas, we found statistically significantly higher cumulative BEWE scores than in rural areas (p = 0.0058).

**Table 1:** Mean values of the BEWE cumulative scores by geographic location in Hungary

Urban			Rural		
Location	Number	Mean (SD)	Location	Number	Mean (SD)
Budapest	66	0.63 (1.03)	Gödöllő	27	0.15 (0.46)
Győr	45	0.16 (0.52)	Tét	28	0.14 (0.52)
Pécs	49	0.71 (1.12)	Boly	51	0.45 (0.88)
Szombathely	19	1.21 (1.31)	Kiskunfélegyháza	42	0.71 (0.94)
Szeged	26	0.46 (0.81)	Szikszó	45	0.29 (0.66)
Miskolc	55	0.36 (0.68)	Józsa	46	0.22 (0.76)
Nyiregyháza	29	0.06 (0.37)	Tiszavasvári	51	0.04 (0.28)
Total	289	0.48 (0.92)*		290	0.29 (0.72)*

p = 0.0058. Total: urban + rural: 0.39 (0.83).

10.5% of the maxillary teeth and 13.8% of the mandibular teeth were affected, but this difference was not statistically significant. Analysing the BEWE scores by oral location, the averages for mandible and maxillar were 0.14 ± 0.45 and 0.25 ± 0.64 respectively, and the difference was statistically highly significant (p < 0.001). Only scores of 0 and/or 1 were found in the maxilla (1st, 2nd and 3rd sextant) and in the mandibular molars (4th and 6th sextant) while in the 5th sextant, which includes the four mandibular incisors (33-43), a BEWE score of 2 was found in two cases (0.3%). The percentage of sextants affected are shown in Table 2. Interestingly enough, most of the participants had a bilaterally symmetrical pattern for their BEWE scores.

**Table 2:** Prevalence of BEWE scores in the examined sextants

Sextant (teeth examined)		1st sextant (16)	2nd sextant (12, 11, 21, 22)	3rd sextant (26)	All upper sextants
BEWE score	0	97%	92.4%	96.8%	95.4%
	1	3.0 %	7.6 %	3.2 %	4.6%
	2	0.0 %	0.0 %	0.0 %	0.0 %

BEWE score	0	88.9%	98.1%	88.9%	91.97%
	1	11.1 %	1.6 %	11.1 %	7.93%
	2	0.0	0.3 %	0.0	0.1 %
Sextant (teeth examined)		6th sextant (46)	5th sextant (42, 41, 31, 32)	4th sextant (36)	All lower sextants

BEWE scores: 0: no erosive tooth wear; 1: initial loss of surface texture; 2: distinct defect, hard tissue loss <50% of the surface area.

The highest percentage of mild erosion was found on the mandibular molars (11.6% and 11.1% on the left and right sides, respectively) followed by 7.6% on the maxillary incisors. The lowest scores were found for the maxillary molars (3.0% and 3.2% on the right and left side, respectively) and 1.9% (BEWE 1 and 2 combined) in the 5th sextant. No dentinal erosion was observed on any of the teeth examined.

### Explanatory Factors

A statistically significant correlation (p = 0.034) was observed between the prevalence of erosion and the daily consumption of erosive drinks (cola and other carbonated drinks). Children consuming these types of beverages at least once daily had statistically significantly more frequent total BEWE scores ≥ 3, compared to those consuming these drinks less often (16.4% vs 10% respectively; p = 0.034) (Table 3).

**Table 3:** Association between the consumption of carbonated soft drinks and BEWE scores

	BEWE score	BEWE 0–2	BEWE > 2	Total n
Consumption of carbonated soft drinks	Once or more daily % (n)	83.6% (178)	16.4% (35)*	213
	Less often than daily % (n)	90% (305)	10% (34)*	339
	Total n	483	69	552**

*Fisher’s 2-sided Exact Test, statistical significance p = 0.034. **Not all of the questions were answered by all of the participants, hence the differences in participant numbers.

The educational level of the mothers also showed a statistically significant correlation with the severity of erosion. The total BEWE score of ≥ 3 was seen statistically significantly more often in children whose mothers did not have a university degree compared to those who did (22.5% vs 8.4% respectively; p = 0.000) (Table 4). Other associations examined, such as between dental erosion and toothbrushing frequency, fresh fruit consumption, consumption of non-carbonated fruit juices, tea with sugar, sweet candies and chocolate, were not statistically significant (Table 5.).

**Table 4:** Association between the educational level of the mother and BEWE scores

	BEWE score	BEWE 0-2	BEWE>2	Total n
Educational level of the mother	Highschool graduate or lower % (n)	77.5% (76)	22.5% (22)*	98
	University degree % (n)	91.6% (281)	8.4% (26)*	307
	Total n	357	48	405**

**Fisher’s 2-sided test; statistical significance p = 0.000. **Not all of the questions were answered by all of the participants, hence the differences in participant numbers.

**Table 5:** Association between possible influencing factors of erosion and BEWE scores 0 to 2, and >2

Related factor		BEWE 0-2	BEWE>2	Total	Fisher’s 2-sided exact test, p-value
Number of daily toothbrushing	Max. 1 per day	214	33	247	0.605
	Two or more per day	273	36	309	
Consumption of fresh fruits	At least once daily	354	51	405	1.000
	Less frequently than daily	127	18	145	
Tea with sugar	At least once daily	250	37	287	0.898
	Less frequently than daily	226	32	258	
Non-carbonated fruit juices	At least once daily	269	40	309	0.354
	Less frequently than daily	208	27	235	
Sweets, candys	At least once daily	271	44	315	0.096
	Less frequently than daily	209	23	232	

Statistical significance set at p < 0.05.

## Discussion

Tooth surface loss, and especially erosive tooth wear, is a growing problem that has received considerable attention. Unfortunately, in recent decades, many indices have been proposed,^2,4^ and the variability of the assessments have made comparisons between studies very difficult.^6^

We decided to use a straightforward, reliable, internationally accepted index in our erosion study: the BEWE index. It is a valuable tool for general practice and allows comparison of data obtained by different examiners. Furthermore, the final BEWE score can be classified and matched to risk levels and it also provides a practical guide to the clinical management of patients.^2^

Our prevalence data reveal a considerable percentage (21.2 %) of children with dental erosion in Hungary. It was not possible to compare our results to previously published Hungarian studies, since no similar examinations had been performed before.

Our prevalence results in this age group are lower compared to most previously published surveys from other countries, which found erosion prevalence to range between 28.3% and 96%.^3-5,7,9,11,14,21^ On the other hand, other authors found even lower prevalences, from 13% to 18.6%.^12,13,20^ The present data fall on the lower part of the scale of the data above. The reasons behind these enormous differences in prevalence are unclear, but they surely cannot be explained by only one factor. Some national traditional cuisines include more acidic food than others, which might be a factor in the differences. Besides cultural and dietary factors, differences in personal oral hygiene habits, as well as professional dental fluoridation or fluoride content of drinking water, probably also play an important role. Finally, the different indices used in different studies also make comparison of the data difficult. Our hypothesis, that the prevalence of dental erosion in Hungarian among 12-year-olds would be similar to the European average, was rejected. In our study, the type of erosion was exclusively enamel loss; no erosion-exposed dentin was observed. In contrast, some of the previous publications reported dentin exposure.^3,4,12,20^

No statistically significant gender differences were observed either in the prevalence or in the severity of erosion in our study. Gender as an independent variable is also controversial, as some of the studies did find a correlation^3,4,9^ and some of them did not.^4,5,7,11-14,20,21^ Interestingly, if a difference was detected, boys had a higher prevalence of dental erosion in this age group. This might be connected to dietary habits, such as consumption of carbonated soft drinks, which is one of the most important risk factors.^5,7,9,11,13,21^ In this age group, girls might drink less of these beverages to reduce caloric intake. A detailed comparison of the results from different studies are summarised in Table 6.

**Table 6:** Overwiew of erosion studies among adolescents

Study	Publication date	Examination location	Age	Index	Questionnaire	Erosion prevalence	Dentin exposure	Gender differences	Carbonated soft drinks	Socioeconomic differences
Our data	2021	Hungary	12 years	BEWE	Yes	21.2%	No	No	Significant correlation	Mother’s education Primary or lower: 99% Secondary or higher: 86%
Dugmore and Rock et al^3^	2004	Leicestershire, UK	12 years	Own	No	59.7%	2.7%	Boys 63.9% Girls 55.3%	Not investigated	Between Caucasians Privileged: 60% Intermediate: 67.5% Deprived: 67%
El Aidi et al^4^	2008	Netherlands	12 years 1.5 years later	Lussi erosion	No	32.2% 42.8%	0% 2.6%	No More boys	Not investigated	SES group low: 36.8% medium: 29.5% high: 28.9%
El Karim et al^5^	2007	Khartoum Sudan	12–14 years	Smith and Knight	Yes	66.9%	21.7%	No	Statistically significant correlation	Private school: 86.6% Public school: 47.5%
Gonzalez-Aragon Pineda et al^7^	2020	Mexico City, Mexico	12 years	BEWE	Yes	62.5%	No	No	Statistically significant correlation	Not investigated
Jarkander et al^9^	2017	Stockholm county, Sweden	15 years 17 years	SEPRS	Yes	28.3% 34.3%	male 22.3% female 14.4%	Boys 34.4% Girls 28.2%	Statistically significant correlation	Not investigated
Maharani et al^11^	2019	Jakarta Indonesia	12 years	BEWE	Yes	96%	Not mentioned	No	Statistically significant correlation	Mother’s education Primary or lower: 99% Secondary or higher: 86%
Peres et al^12^	2005	Brasil	12 years	O’Sullivan	No	13%	0.32%	No	Not investigated	Private school: 78.9% Public school: 90.3%
Provatenou et al^13^	2016	Thessaloniki Greece	8 years 14 years	BEWE	Yes	14.6% 21%	No†	No†	Statistically significant correlation	Not investigated
Sanhouri et al^14^	2010	Khartoum Sudan	14 years	Own	No	74%	22.9%	No	No statistically significant differences	No significant differences
Zhang et al^20^	2015	Central China	12 year 15 years	BEWE (modified)	Yes	18.6%	1.9%	No	Drinking habits	Socioeconomic class: low 58.7% high: 51%
Zhang et al^21^	2014	Hong Kong	12 years	BEWE	Yes	75%	No	No	Statistically significant correlation	Not investigated

†On permanent teeth

In our results, mandibular teeth had higher BEWE scores than did maxillary teeth (0.14 ± 0.45 vs 0.25 ± 0.64, respectively p < 0.001), which is in contrast to previous findings, where more erosive signs were reported in maxillary (66.7%) than in mandibular teeth (51.7%).^14^ In that study,^14^ the palatal surfaces of the maxillary incisors were the most affected sites, followed by the occlusal surfaces of the mandibular molars, which were most affected in our study. Another study found a predominance of erosion on the occlusal surfaces of the molars and the palatal surfaces of the maxillary anterior teeth.^4^ A midline symmetry of erosion was found in our study that was consistent with previous findings.^6,18^

On the basis of our results, it can be concluded that most Hungarian children have no risk of erosion (BEWE score 0-2) and only 2.3% of them could be considered low risk (BEWE score 3-8), judging by the BEWE scores. For these children, oral hygiene and dietary assessment and advice, as well as routine maintenance and observation repeated at 2-year intervals, are suggested by the BEWE guide for clinical management.^2^ The present nationwide erosion prevalence data indicate that more attention must be given to children at risk and also to those who live in risk areas. It is obvious from our results that there are considerable differences in erosion level between different geographic locations in Hungary (Table 1), but the reasons behind this pattern are unclear. Higher scores were mostly observed in urban areas, while the lowest scores were seen in some rural regions. However, independent of the location, there might be other factors influencing erosion development, such as dietary habits and the fluoride concentration of the water. It would be necessary to perform a more detailed analysis of these external factors influencing erosion in Hungary.

In our study, we found a statistically significant correlation (p < 0.034) between the prevalence of erosion and the daily consumption of erosive drinks (cola and other carbonated soft drinks), which is in accordance with previously published data.^5,7,8-11,13,21^

Currently, there is no agreement in the literature regarding the correlation between prevalence and/or severity of dental erosion and socioeconomic factors. However, we detected a statistically significant correlation (p = 0.000) between the severity of erosion and the educational level of the mother. This is similar to previous studies, which found more tooth surface loss among children in lower socioeconomic groups.^3,4,8,11,12,20^ On the other hand, according to another study, there was no statistically significant correlation between the parents’ educational level and dental erosion in their children.^14^ Furthermore, there are data reporting higher erosion prevalence between adolescents visiting private school vs public school.^5^ Dietary factors, first of all acidic drinks, seem to play a crucial role in the development of erosion. There might be major differences in the consumption of these erosive drinks between the socioeconomic groups, which is also different from country to country. In European studies, we see lower erosion prevalence correlated with higher socioeconomic level. This might be a result of the more health-conscious diet in this population. In contrast, in developing countries, lower socio-economic status seems connected with less erosion. In these countries, one possible reason for this reverse pattern is that the poorer population cannot afford these more expensive, erosive soft drinks. Nowadays several highly erosive drinks are available on the market, such as energy drinks, ice tea and carbonated soft drinks.^10^ The consumption of these beverages is probably very heterogeneous between the different socioeconomical groups, which might play an important role in these differences.

## Conclusions

We found an erosion prevalence of 21.2% among 12-year-old Hungarian adolescents, which is lower when compared to previously reported Western-European and Asian data. The severity of erosion was also lower in our study than reported earlier. Gender was not a statistically significant determining factor. There were considerable differences, however, in the erosion level between geographic locations and location types. A strong association between erosion and the daily consumption of erosive drinks, as well as between erosion and the educational level of the mother, was shown.

## References

[ref1] Bardolia P, Burnside G, Ashcroft A, Milosevic A, Goodfellow SA, Rolfe EA, Pine CM (2010). Prevalence and risk indicators of erosion in thirteen- to fourteen-year-olds on the Isle of Man. Caries Res.

[ref2] Bartlett D, Ganss C, Lussi A (2008). Basic erosive wear examination (BEWE): A new scoring system for scientific and clinical needs. Clin Oral Investig.

[ref3] Dugmore CR, Rock WP (2004). The prevalence of tooth erosion in 12-year-old children. Brit Dent J.

[ref4] El Aidi H, Bronkhorst EM, Truin GJ (2008). A longitudinal study of tooth erosion in adolescents. J Dent Res.

[ref5] El Karim IA, Sanhouri NM, Hashim NT, Ziada HM (2007). Dental erosion among 12-14 year old school children in khartoum: A pilot study. Community Dent Health.

[ref6] Ganss C, Lussi A (2008). Current erosion indices –flawed or valid?. Clin Oral Investig.

[ref7] González-Aragón Pineda ÁE, Borges-Yáñez SA, Lussi A, Aguirre-Hernandez R, García-Pérez Á (2020). Prevalence, incidence, and progression of erosive tooth wear and their respective risk factors among schoolchildren in Mexico City. Pediatr Dent.

[ref8] Hasheminejad N, Mohammadi TM, Mahmoodi MR, Barkam M, Shahravan A (2020). The association between beverage consumption pattern and dental problems in Iranian adolescents: a cross sectional study. BMC Oral Health.

[ref9] Jarkander MS, Grindefjord M, Carlstedt K (2018). Dental erosion, prevalence and risk factors among a group of adolescents in Stockholm County. Eur Arch Paediatr Dent.

[ref10] Lussi A, Jaeggi T, Lussi A (2006). Dental erosion in children. Monographs in Oral Science.

[ref11] Maharani DA, Zhang S, Gao SS, Chu C-H, Rahardjo A (2019). Dental caries and the erosive tooth wear status of 12-year-old children in Jakarta, Indonesia. Int J Environ Res Public Health.

[ref12] Peres KG, Armenio MF, Peres MA, Traebert J, De Lacerda JT (2005). Dental erosion in 12-year-old schoolchildren: A cross-sectional study in Southern Brazil. Int J Paediatr Dent.

[ref13] Provatenou E, Kaklamanos EG, Kevrekidou A, Kosma I, Kotsanos N (2016). Erosive tooth wear and related risk factors in 8- and 14-year-old greek children. Caries Res.

[ref14] Sanhouri NM, Ziada HM, Ahmed GI (2010). Kamis AH Tooth surface loss, prevalence and associated risk factors among 12-14 years school children in Khartoum State, Sudan. Comm Dent Health.

[ref15] Szőke J, Petersen PE (2000). Evidence for dental caries decline among children in an East European country (Hungary). Commy Dent Oral Epidemiol.

[ref16] Tantbirojn D, Pintado MR, Versluis A, Dunn C, Delong R (2012). Quantitative analysis of tooth surface loss associated with gastroesophageal reflux disease: A longitudinal clinical study. JADA.

[ref17] Truin GJ, van Rijkom HM, Mulder J, van’t Hof MA (2005). Caries trends 1996-2002 among 6- and 12-year-old children and erosive wear prevalence among 12-year-old children in the Hague. Caries Res.

[ref18] Truin GJ, van Rijkom HM, Mulder J, van’t Hof MA (2004). Dental caries and dental erosion among 5- and 6-year old and 11- and 12-year old school children in the hague, the netherlands. Changing prevalences. Nederlands Tijdschrift voor Tandheelkunde.

[ref19] World Health Organization (2013). Oral Health Surveys: Basic Methods.

[ref20] Zhang J, Du Y, Wei Z, Tai B, Jiang H, Du M (2015). The prevalence and risk indicators of tooth wear in 12- and 15-year-old adolescents in Central China. BMC Oral Health.

[ref21] Zhang S, Chau MA, Lo ECm, Chu C-H (2014). Dental caries and erosion status of 12-year-old Hong Kong children. BMC Public Health.

